# Hepatoprotective Potential of Pomegranate in Curbing the Incidence of Acute Liver Injury by Alleviating Oxidative Stress and Inflammatory Response

**DOI:** 10.3389/fphar.2021.694607

**Published:** 2021-11-26

**Authors:** Hamid Ali, Azra Jahan, Samrana Samrana, Abid Ali, Safdar Ali, Nurul Kabir, Amjad Ali, Riaz Ullah, Ramzi A. Mothana, Bibi Nazia Murtaza, Muhammad Kalim

**Affiliations:** ^1^ Department of Biosciences, COMSATS University, Islamabad, Pakistan; ^2^ Department of Zoology, Abdul Wali Khan University, Mardan, Pakistan; ^3^ College of Agriculture and Biotechnology, Zhejiang University, Hangzhou, China; ^4^ Department of Physics, University of Swabi-Anbar, Mardan, Pakistan; ^5^ Faculty of Science, Institute of Biological Sciences, University of Malaya, Kuala Lumpur, Malaysia; ^6^ Faculty of Biological Sciences, Department of Biochemistry, Quaid-i-Azam University, Islamabad, Pakistan; ^7^ Department of Pharmacognosy, College of Pharmacy, King Saud University, Riyadh, Saudi Arabia; ^8^ Department of Zoology, Abbottabad University of Science and Technology, Abbottabad, Pakistan; ^9^ Cancer Research Institute, Houston Methodist Hospital, Houston, TX, United States

**Keywords:** pomegranate, hepatotoxicity, histology, oxidative stress, Kupffer cells, iNOS

## Abstract

Hepatitis is an inflammatory disease of the liver and is considered one of the leading causes of death worldwide. Due to its scavenging activity, *Punica granatum* may be used for the treatment and prevention of liver diseases. The current study investigated the protective mechanism underlying the effects of pomegranate against a rat model of carbon tetrachloride–induced liver injury. Intraperitoneal injection of CCl_4_ resulted in liver inflammation, oxidative stress, and accumulation of lipid in hepatocytes. CCl_4_ induced a downregulation of superoxide dismutase (SOD), glutathione (GSH), and melonaldehyde (MDA). Pomegranate protection was assessed in terms of biochemical parameters, histopathology, and immunohistochemistry. Promegranate administration decreased inflammation, elevated serum enzymes and ROS production, and countered the debilitating effects caused by CCl_4_. In addition, CCl_4_-induced histological changes were absent in the crude pomegranate extract group, which also enhanced the scavenging activity of reactive oxygen species by enhancing the antioxidant defense mechanism as confirmed by detecting MDA, SOD, and GSH expressions. The migration of CD68^+^ macrophages was halted at the injured area of the central vein and the number of macrophages was reduced to the normal control by the crude extract compared to the positive control silymarin group. Likewise, protective effects of ethylacetate and the aqueous fraction of the crude extract were also observed. However, the butanol and *n*-hexane fractions displayed increased levels of ALT, AST, and ALP as compared to silymarin. About 25% damage to hepatocytes was observed in the butanol and *n*-hexane group by histopathological examination, which is a little better compared to the CCl_4_-treated group. The crude extract and its ethyl acetate and aqueous fractions may be accountable for the hepatoprotective potential of *Punica granatum*, which was further confirmed by *in vivo* experiments. Together, these findings confirm that pomegranate exerts hepatoprotective activity against CCl_4_-induced oxidative stress and liver damage.

## Introduction

Liver diseases are one of the most serious health issues in the world. The liver is concerned with metabolism and detoxification of exogenous substances such as drugs, toxic chemicals, and viruses and hence is very prone to injury (Moore et al., 2010; Moore et al., 2013). Toxic chemicals are the most serious cause of liver injury, mediated by the free radical–induced oxidative stress (Rechnagel et al., 1973). Carbon tetrachloride (CCl_4_) is one of the toxic xenobiotics widely used in animal models to induce oxidative stress–mediated hepatitis (Pierce et al., 1987), resulting in apoptosis, inflammation, and fibrosis (Wong et al., 1998). The main mechanism through which CCl_4_ induces hepatotoxicity is the generation of free radicals (Qureshi et al., 2009). CCl_4_ is metabolized by the cytochrome P450 enzymes to a highly reactive trichloromethyl (CCl_3_^−^) free radical which reacts with free oxygen to form the trichloromethyl peroxy radical (CCl_3_OO^−^). The free radical sequentially attacks the hepatocyte cell membrane, leading to lipid peroxidation and cell death. The damaged hepatocytes are linked with elevated serum levels of ALT, AST, and ALP and depletion of CAT, GSH, and SOD activities (Chen et al., 2017). In addition, CCl_4_ intoxication triggers the production of proinflammatory cytokines such as TNF-*α*, TGF-*β*, and IL-6, stimulating inflammatory cell recruitment at the site of injury (Sun et al., 2001).

Naturally, the body’s non-enzymatic or enzymatic antioxidant defenses can inhibit the injurious effects of toxic agents through free radical scavenging activity or by modulation of the inflammatory response (Percival, 1998; Domitrovic et al., 2009). However, when free radical generation overwhelms the antioxidants, oxidative damage of the hepatic cells follows (Bandyopadhyay et al., 2002). It has been reported that intake of a nonspecific antioxidant-rich diet is often linked with the risk of developing different liver diseases due to their uncertain formulation and unknown mechanism of action. Therefore, effective antioxidants and anti-inflammatory remedies with a clear mechanism of action and no side effects are urgently required for the treatment of liver diseases (Praneetha et al., 2019).

The modern pharmaceutical industry is still struggling to design reliable hepatoprotective drugs. Therefore, a large number of traditional remedies can be suggested as alternatives for the treatment of liver ailments. Many plant species have been used traditionally for the treatment of liver disorders around the globe such as *Verbena litoralis, V. montevidensis* (Vestena et al., 2019), *Boerhaavia diffusa* (Olaleye et al., 2010), *Annona squamosa*, *Cymbopogon citratus* (Rajeshkumar et al., 2015), *Silybum marianum* (AI Jarallah et al., 2013), *Fumaria indica* (Rathi et al., 2008), and *Punica granatum* (Stowe, 2011). These plants contain a wide range of bioactive constituents such as steroids, terpenoids, phenols, and flavonoids, exhibiting hepatoprotective activities.

The pomegranate (*Punica granatum*), commonly known as anar, a deciduous tree belonging to the Punicaceae family, is found all over Pakistan. In the holy book (Al-Qur’an) of the Muslims, it is mentioned as the fruit of “Adan” (Heaven). The pomegranate is one of the healthiest fruits on this planet (Malik et al., 2005). All of its parts contain beneficial compounds, and hence, it is consumed in many disease conditions globally. The flowers of the pomegranate have significant hepatoprotective and anti-inflammatory potential (Kaur et al., 2006; Stowe, 2011) and may also control glucose levels (Huang et al., 2005). The nephro-protective potential of the pomegranate has been credited to its seed oil (Bouroshaki et al., 2010). Recent studies have suggested that pomegranate (PG) derivatives abate chemical-induced skin, breast, lung, and colon cancer (Adhami et al., 2009). Fruit and fruit extracts of PG have been used for the last decade, as anticipatory and antioxidant agents against several life-threatening diseases such as type 2 diabetes (Banihani et al., 2013), cancer (Orgil et al., 2014), and cardiovascular diseases (Al-Jarallah et al., 2013; Hamoud et al., 2014). Nutraceutical properties of the PG fruit are not only restricted to the comestible part (arils) but also to the nonedible parts (peel and seed) which may appear to be useless, but actually contain higher amounts of medicinally and nutritionally significant phytochemicals than the comestible part (Abid et al., 2017). The PG peel (PP), constituting nearly 50% of the weight of the fruit, is characterized by different types of high–molecular weight antioxidant compounds present in it that have attracted many researchers to further explore its beneficial aspects (Hong et al., 2015).

PP and PPx (pomegranate peel extract) have been reported for substantial hepato-protection by attenuating inflammatory responses (Bchoual et al., 2011; El-Rashedy et al., 2011). Particularly, a rich variety of PG phenolic compounds such as flavonoids, phenolic acids (Soliman and Selim, 2012; Singh et al., 2017), and tannins has been reported for the hepatoprotective activity against fatty liver disorder (El-Rashedy et al., 2011). Among these compounds, flavonoids are the ones which have a considerable variety of activities such as anti-inflammatory, antidiabetic, anti-allergic, antiplatelet, anti-mutagenic, and antioxidant activities (Xiao, 2017; Khan et al., 2018).

Taking into account the above points, this work was designed to investigate the hepatoprotective, free radical–quenching, and anti-inflammatory activities of PPx and its fractions in Wistar rats, thereby providing scientific contribution for the incorporation of herbal remedies with modern medicine.

## Materials and Methods

### Plant Material

Ripe and healthy pomegranate fruits were collected from the northern parts of Khyber Pakhtunkhwa and authenticated by the taxonomist Dr. Muhammad Ilyas at the Department of Botany, University of Swabi, Pakistan, with the voucher number (PG/HA-1), deposited in the herbarium.

### Extract Preparation

**FIGURE 1:**
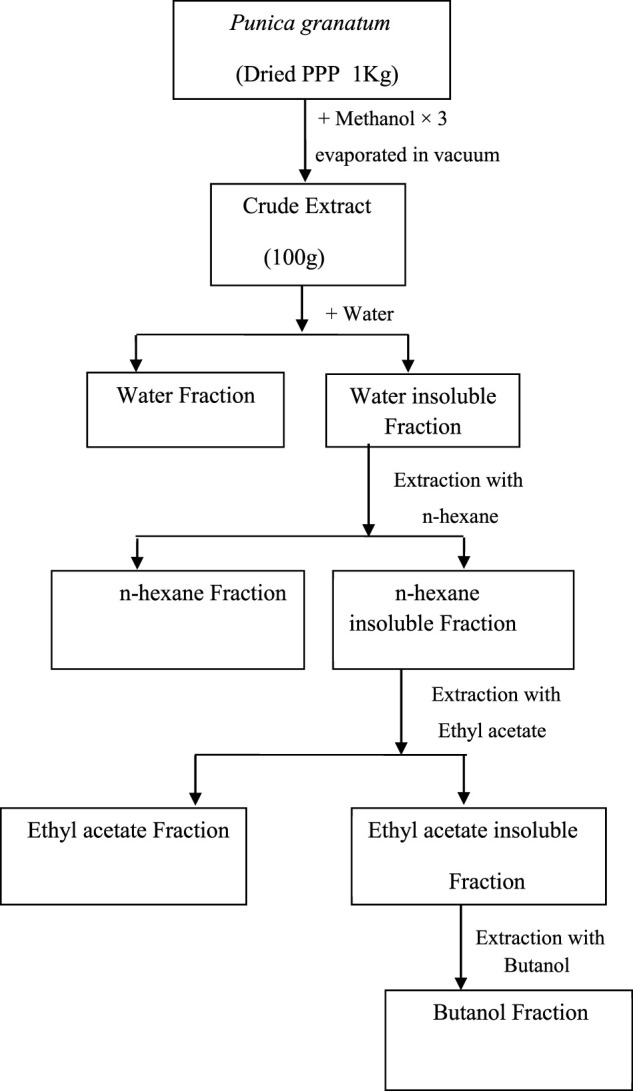
Preparation of extracts from *Punica granatum* peel powder (PPP).

Pomegranate fruits were collected, washed thoroughly with tap water, and then peeled. The peels were shade-dried for one month. The dried peels were powdered in an electric grinder. The accurately weighed powdered sample (1 kg)/was mixed in 2 L of methanol to yield 100 g extract (Met-PPx). After removal of the solvent, the concentrated methanolic extract was suspended in water for further solubilization to get the aqueous fraction (15 g). Then *n*-hexane was added to the mixture to remove the fatty material, and the defatted fraction was then dissolved in ethylacetate and butanol to finally yield *n*-hexane (12 g), ethylacetate (25 g), and butanol (9 g) fractions, respectively. The extract and fractions were kept at 4°C until further use. The extract preparation scheme is given below in Figure 1.

### Animals

Male Wistar rats (150–180 g) were housed in individual cages for a week at ambient temperature under 12-h light/dark cycles, with access to chow and tap water *ad libitum* according to standard laboratory protocol. All animals received humane care, and all protocols concerning the animals were in agreement with the guidelines approved by the Institutional Ethics Committee approval letter (CUI/Bio/ERB/4-21/19) of COMSATS University, Islamabad.

### CCl_4_-Induced Acute Hepatotoxicity

Acute hepatotoxicity studies were performed using male Wistar rats. The experimental protocol for the current study was based on previous studies (Ali et al., 2014). All the experimental animals were divided into different groups, and six rats were included in each group as described here: Group 1 (normal control) were injected with vehicle only (1 ml/kg body weight olive oil), Group 2 (hepatotoxicity group) were treated with i.p. injection of CCl_4_ (1 ml/kg) with 1:1 olive oil, Group 3 (positive control) were injected intraperitoneally with CCl_4_ (1 ml/kg) with 1:1 olive oil and also administered orally with silymarin (100 mg/kg) 3 days before treatment and 2 days after treatment, and Groups 4–8 were treated with i.p. injection of CCl_4_ (1 ml/kg) with 1:1 olive oil but also received methanolic extract, aqueous, *n*-hexane, ethyl acetate, and butanol fractions, respectively, at a dose of 100 mg/kg body weight for 3 days before treatment and 2 days after treatment.

### Blood Biochemistry

All experimental animals were terminated 48 h after the administration of CCl_4_ under sodium pentothal anesthesia. From animals, the blood was collected by cardiac puncture using a 5-ml sterile syringe and with a slight possible pressure to avoid hemolysis. Blood was centrifuged for 15 min at 2000 rpm to extract serum. The serum was used for the determination of liver damage by analyzing biological parameters (ALT, AST, and ALP) using a dry chemistry analyzer (Roche Diagnostics, Mannheim, Germany).

### Oxidative Stress Markers

For identification of oxidative stress, the livers were excised and homogenized (Polytron homogenizer) in 50 mM phosphate buffer saline with a pH value of 7.4 (Kinematica, Lucerne, Switzerland). The homogenates were centrifuged at 15 000 g for 20 min at 4°C using a Beckman L7-65 Ultracentrifuge (Beckman, Fullerton, United States), and the supernatants were used to determine the Cu/Zn SOD activity, MDA, and GSH content. The quantification of protein in liver tissues was assessed using Bradford’s method (Bradford, 1976). The activity of Cu/Zn SOD was measured at 550 nm by the reduction in cytochrome c by superoxide radicals using a UV–VIS spectrophotometer (Domitrović et al., 2011). Similarly, the MDA and GSH contents were determined according to a reported study (Anderson, 1985). The supernatant was deproteinized with 1.2 M metaphosphoric acid and centrifugation at 4500 g for 8 min (Rotina 420R, Andreas Hettich GmbH, Tuttlingen, Germany). Consequently, 700 μL of 0.3 mM NADPH in phosphate buffer saline and 25 μL of the deproteinized sample along with water and 100 μL of 6 mM DTNB were thoroughly mixed in a cuvette to get a final volume of 1.0 ml. Finally, 10 μL of glutathione reductase (50U ml^−1^) was added to the mixture, and absorbance was observed for 30 min at 405 nm. From the series of dilution of stock solution of glutathione, their content was identified from the standard curve.

### Histopathological Study of the Liver

After dissection, the liver was identified in the right upper quadrant and sliced up. The liver was kept in formalin for histopathological examination. Formalin-fixed liver tissues were desiccated through a series of graded alcohol, followed by their entrenchment in paraffin, and cut into 6-μm-thick sections. The liver sections were transferred onto the slides, dewaxed, and rehydrated through xylene and graded alcohol in the reverse sequence from dehydration. The deparaffinized tissues were stained with hematoxylin–eosin (H&E) and examined under a bright field microscope. The necrotic area of each group was measured in at least 30 different tissue sections of the liver using NIS-elements software from Nikon, Japan, and was expressed as the percentage of the entire area of the section.

### Immunostaining

The previously dewaxed and rehydrated liver sections (6 μm thick) were used for immunohistochemical assessment. All selected liver sections were incubated with blocking solution in phosphate buffered solution (PBS, Roti-immunoblock, Carl Roth, Karlsruhe, Germany) for 25 min at normal temperature. Subsequently, these tissues were incubated with primary monoclonal antibody for CD68 (clone ED1, abcam) and iNOS (ab3523) at dilution (1:100) for 45 min at 37°C. The liver tissues were thoroughly washed with PBS and incubated with secondary antibodies, FITC/Texas Red-conjugated goat anti-mouse IgG (1:100), for 40 min. After briefly rinsing twice with PBS, the nuclei were stained with DAPI for around a minute and then rinsed with PBS and mounted in mounting media. The immunostained tissues were analyzed by fluorescence microscopy, and their images were acquired using a Nikon DXM 1200 C camera using image analysis software AR 3.0 (Nikon, Japan). All the acquiesced images were processed using Adobe Photoshop software. The number of Kupffer cells was counted in different portions surrounding the injured area from their elongated nuclei stained with DAPI using NIS-elements software.

### Data Analysis

Results are expressed as mean ± SD. Total variation present in a set of data was estimated by one-way analysis of variance (ANOVA), followed by Dunnett’s *post hoc* test. *p* < 0.05 and *p* < 0.01 were considered to be significant.

## Results

### Histopathology of the Liver Indicates Better Hepatoprotective Potential of the MeOH Extract of Pomegranate Peel Than That of Silymarin

**FIGURE 2:**
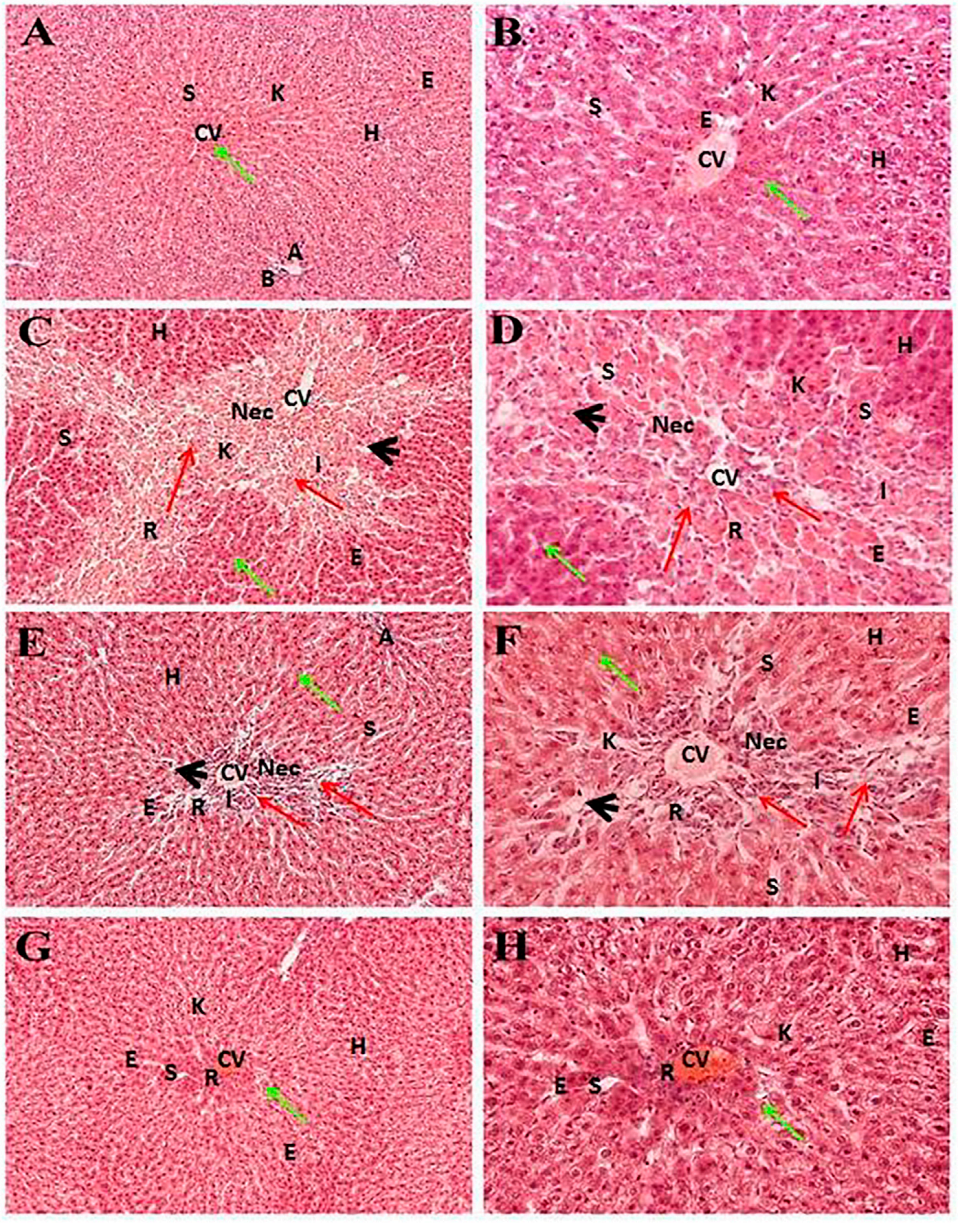
Hepatoprotective potential of Met-PPX. Histological examination of the liver displays a typical (green arrows) central vein in the control group (normal, **(A, B)**); damaged areas (red arrows) after CCl_4_ intoxication (CCl_4_, **(C, D**)); silymarin protection (CCl_4_^+^silymarin **(E, F)**); healing by methanolic extract (CCl_4_^+^ Met-PPx, **(G, H)**). CCl_4_^+^ Met-PPx represented better protection than standard silymarin. Scale bar is 250 and 25 μm, shown in **(G, H)**. CV, central vein; H, hepatocytes; S, sinusoidal spaces; E, endothelial cells; Nec, necrosis; A, artery; R, congestion of RBC; I, infiltration of inflammatory cells; arrowhead, pyknosis; K, Kupffer cells.

**FIGURE 3:**
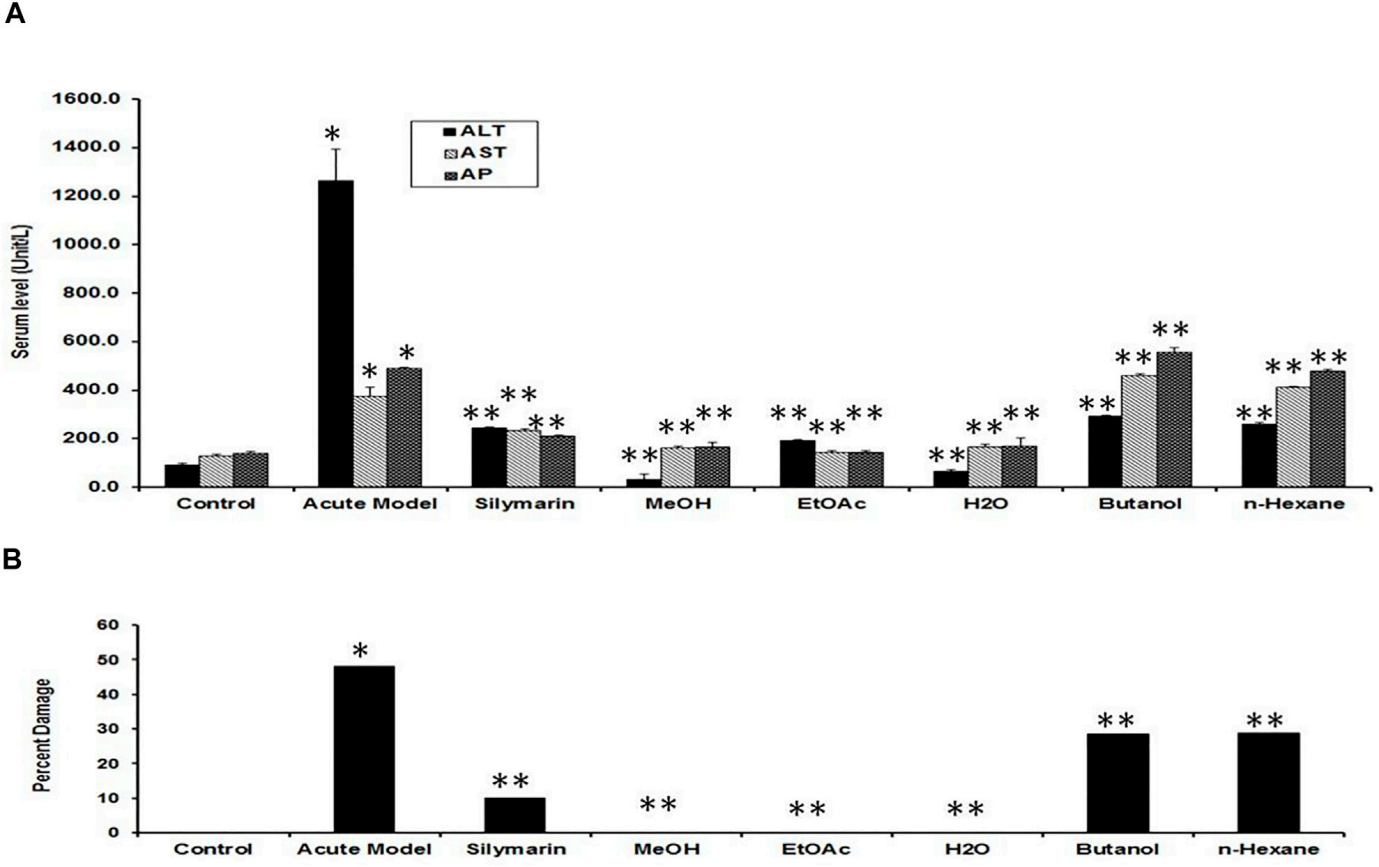
Quantification of the effects of *Punica granatum* peel extract on CCl_4_-induced hepatotoxicity. **(A)** Serum levels of ALT, AST, and ALP act as indicators of hepatic injury. We expressed the data as the mean ± SD where *p < 0.05, when the groups was compared to the normal control; **p < 0.001, when compared to the CCl_4_ group. MeOH extract, EtOAc, and H_2_O fractions showed ample protection against CCl_4_ liver injury (**p < 0.001) compared to CCl_4_+silymarin (**p < 0.001). Other fractions (butanol and *n*-hexane) did not show considerable outcomes. **(B)** Percent hepatic damage under various conditions as evaluated by histology. Note that the silymarin group shows some damage (∼10%), whereas Met-PPx (MeOH), EtOAc, and aqueous (H_2_O) fractions indicated no damage (0%). Effect of PPx on oxidative stress markers.

Liver sections of the normal control group exhibited typical lobular architecture, with a central vein fenced by the hepatic cord of cells with clear spaces lined by elongated endothelial and Kupffer cells (Table 1; Figures 2A,B). In contrast, the CCl_4_-treated group revealed loss of hepatic architecture with clear pale areas having degenerated and necrotic hepatocytes and the presence of inflammatory cells around the central vein with distinct nuclei (Table 1). This pointed toward the fact that the injurious free radical CCl_3_^−^ is particularly generated only in the hepatocytes. Normal-shaped hepatocytes (Figures 2C,D) could also be perceived around the necrotic area, which could simply be identified because of their darker staining characteristic. Cells around the pale necrotic area exhibited hydrophic degeneration and ballooning hepatocytes (Table 1). Moreover, histopathological analysis (Figure 3B) had shown ∼47% damage in the CCl_4_-treated group. Liver samples acquired from the CCl_4_^+^ silymarin–treated group showed a substantially attenuated (*p* < 0.001) necrotic area around the central vein and a great decrease (Figures 2E,F) compared to the CCl_4_ group (Table 1). Moreover, minimal hepatic damage (∼10%) was observed by histopathological analysis (Figure 3B). Interestingly, the CCl_4_^+^ Met-PPx completely prevented liver necrosis (Table. 1; Figures 2G,H) with 0% damage (Figure 3B). Therefore, the Met-PPx at an optimum dose of 100 mg/kg presented restoration of injury and hence showed hepatoprotective activity (*p* < 0.001) compared to the positive control silymarin (100 mg/kg).

**TABLE 1:** Lesion scoring of liver tissue obtained after treatment under different conditions.

Groups	Control	CCl_4_	CCl_4_^+^silymarin	MeOH	H_2_O	EtOAc	Butanol	*n*-hexane
Hepatocellular necrosis	0.00	12.91 ± 0.02^a^	8.12 ± 0.01^a^	2.91 ± 0.11^b^	1.72 ± 0.31^b^	0.10 ± 0.12^b^	9.32 ± 0.06^a^	7.23 ± 0.03^a^
Hepatocellular hypertrophy	0.00	7.2 ± 0.1^a^	5.3 ± 0.3^a^	3.01 ± 0.21^b^	1.11 ± 0.52^b^	0.02 ± 0.52^b^	6.4 ± 0.1^a^	5.8 ± 0.11^a^
Fatty degeneration	0.00	4.21 ± 0.32^a^	2.32 ± 0.11^a^	0.13 ± 0.31^b^	0.21 ± 0.13^b^	0.14 ± 0.21^b^	3.12 ± 0.12^a^	4.32 ± 0.31^a^
Congestion of RBC	0.00	4.1 ± 0.5^a^	3.5 ± 0.2^a^	1.6 ± 0.1^b^	1.2 ± 0.3^b^	0.1 ± 0.2^b^	3.1 ± 0.3^a^	5.2 ± 0.2^a^
Inflammatory cell infiltration	0.00	12.6 ± 0.12^a^	7.4 ± 0.21^a^	1.5 ± 0.12^b^	0.7 ± 0.23^b^	0.3 ± 0.12^b^	9.5 ± 0.11^a^	8.3 ± 0.21^a^
Pyknosis	0.00	6.7 ± 0.25^a^	1.45 ± 0.14^a^	0.35 ± 0.13^b^	0.13 ± 0.21^b^	0.23 ± 0.42^b^	3.6 ± 0.13^a^	2.7 ± 0.11^a^

Treatment effect of pomegranate extract and its fractions on the inflammation scores of liver tissues following CCl_4_-induced hepatotoxicity. The values are means ± SD (*n* = 10), and data were analyzed by one-way ANOVA, followed by Tukey’s *post hoc* test for multiple comparisons.

a Indicates significant from the control group.

b Denotes significant from the CCl_4_ group.

### Hepatoprotective Potential of the EtOAc and Aqueous Fractions of the Met-PPx

**FIGURE 4:**
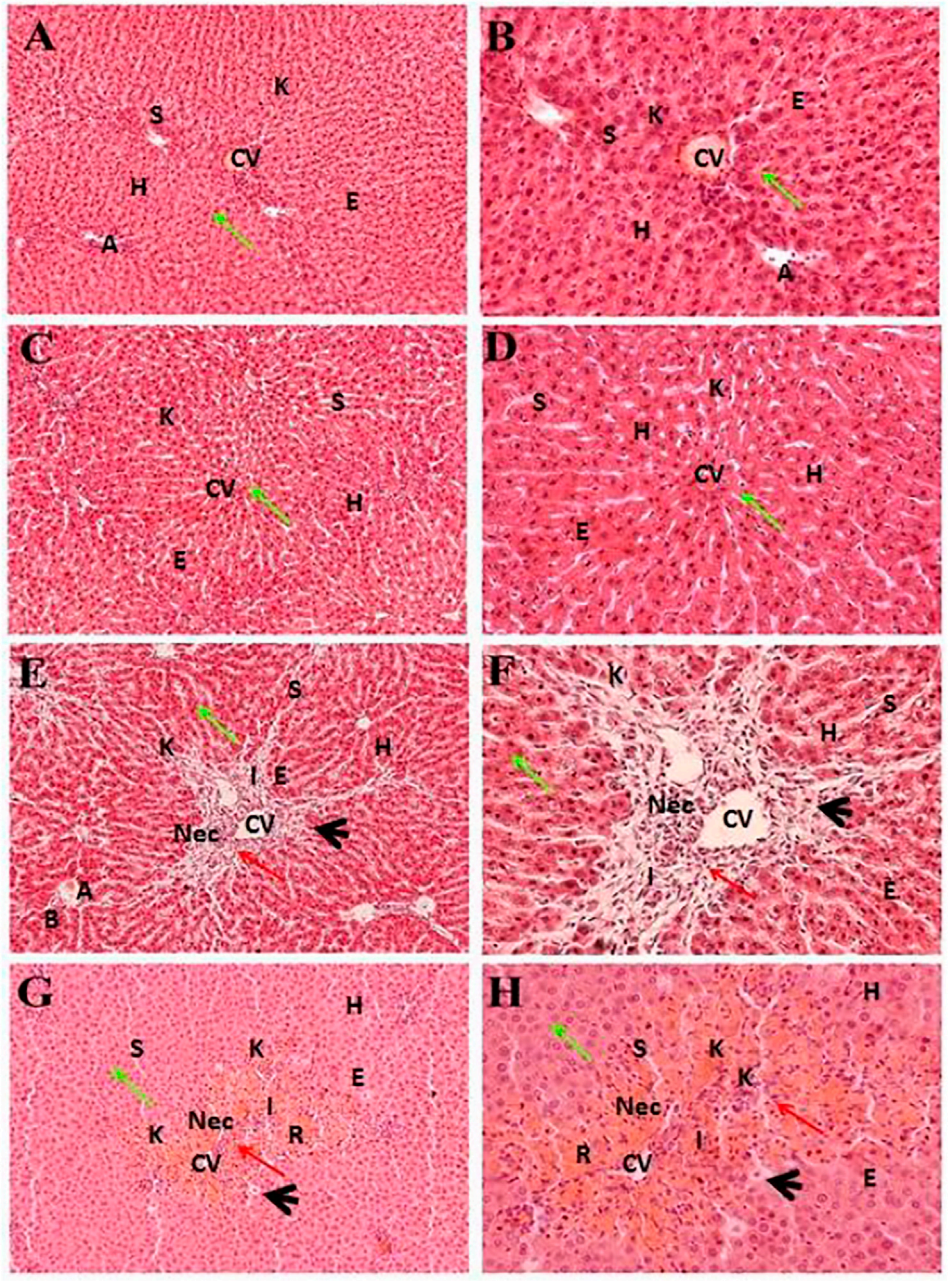
Hepatoprotective potential of different fractions of Met-PPx. Liver histopathology of the ethyl acetate fraction (CCl_4_^+^EtOAc, **(A, B)**) showing normal histology with a prominent central vein. Similar results were seen for the aqueous fraction (CCl_4_^+^H_2_O, **(C, D)**). However, the butanol (CCl_4_^+^Butanol, **(E, F)**) and *n*-hexane (CCl_4_^+^ *n*-hexane, **(G, H)**) fraction groups showed damage to the hepatic architecture (red arrows). CV, central vein; H, hepatocytes; S, sinusoidal spaces; E, endothelial cells; Nec, necrosis; A, artery; R, congestion of RBC; I, infiltration of inflammatory cells; arrowhead, pyknosis; K, Kupffer cells.

Figure 4 indicates the effect of different fractions of the Met-PPx on the histology of CCl_4_-induced liver toxicity. The CCl_4_^+^ EtOAc group had shown a similar protective effect to that of the Met-PPx, with restoration of the hepatic architecture with no sign of necrosis (Table. 1; Figures 4A,B), and presented 0% damage (Figure 3B). Even the liver architecture was in such a way that it was difficult to distinguish it from the untreated group. Likewise, the CCl_4_^+^ aqueous group also completely abolished and showed normal arrangement of hepatocytes with no sign of necrosis (Figures 4C,D) and 0% damage (Table 1; Figure 3B). On contrary, more necrosis and massive inflammatory recruitment (Table 1) around the central vein was found in the CCl_4_^+^ butanol group than in the positive control silymarin (Figures 4E,F). Histopathological investigation revealed ∼29% damage (Figure 3) which is less than that in the CCl_4_-treated group but more than that in the silymarin control. The CCl_4_^+^ *n*-hexane group also indicated necrotic areas (Table 1) around the injurious place of the central vein with congestion of RBCs (Figures 4G,H).

### PPx Alleviated Hepatic Enzyme Level

Several enzymes coming from the liver in the serum were used as useful biochemical markers for investigating early hepatotoxicity. After 48 h of CCl_4_ intoxication, rats demonstrated a substantial increase in the levels of serum ALT, AST, and ALP as compared to the normal control (Figure 3A). The positive control silymarin at a dose of 100 mg/kg was not as effective in the reduction of hepatocellular damage as 100 mg/kg Met-PPx, which reduced (*p* < 0.001) the rise in the serum level of hepatic enzymes to a remarkable extent. However, it is interesting and noteworthy that in the silymarin-treated group, the level of enzymes was not completely halted down to the control, which represents some damage to the hepatocytes. Interestingly, the Met-PPx substantially stopped (*p* < 0.001) the rise in the level of serum enzymes and brought it down to control levels, indicating no damage to the parenchyma cells regardless of the CCl_4_ treatment. The EtOAc fraction abrogated (*p* < 0.001) the elevation in serum enzymes. Similarly, the aqueous fraction also inhibited (*p* < 0.001) the increase in serum enzymes. However, the butanol and the *n*-hexane fractions did not considerably reduce the hepatic enzymes. These results strongly suggest that the hepatoprotective and free radical–quenching potential of the PP exists in the EtOAc and aqueous fraction due to the existence of bioactive compounds.

**FIGURE 5:**
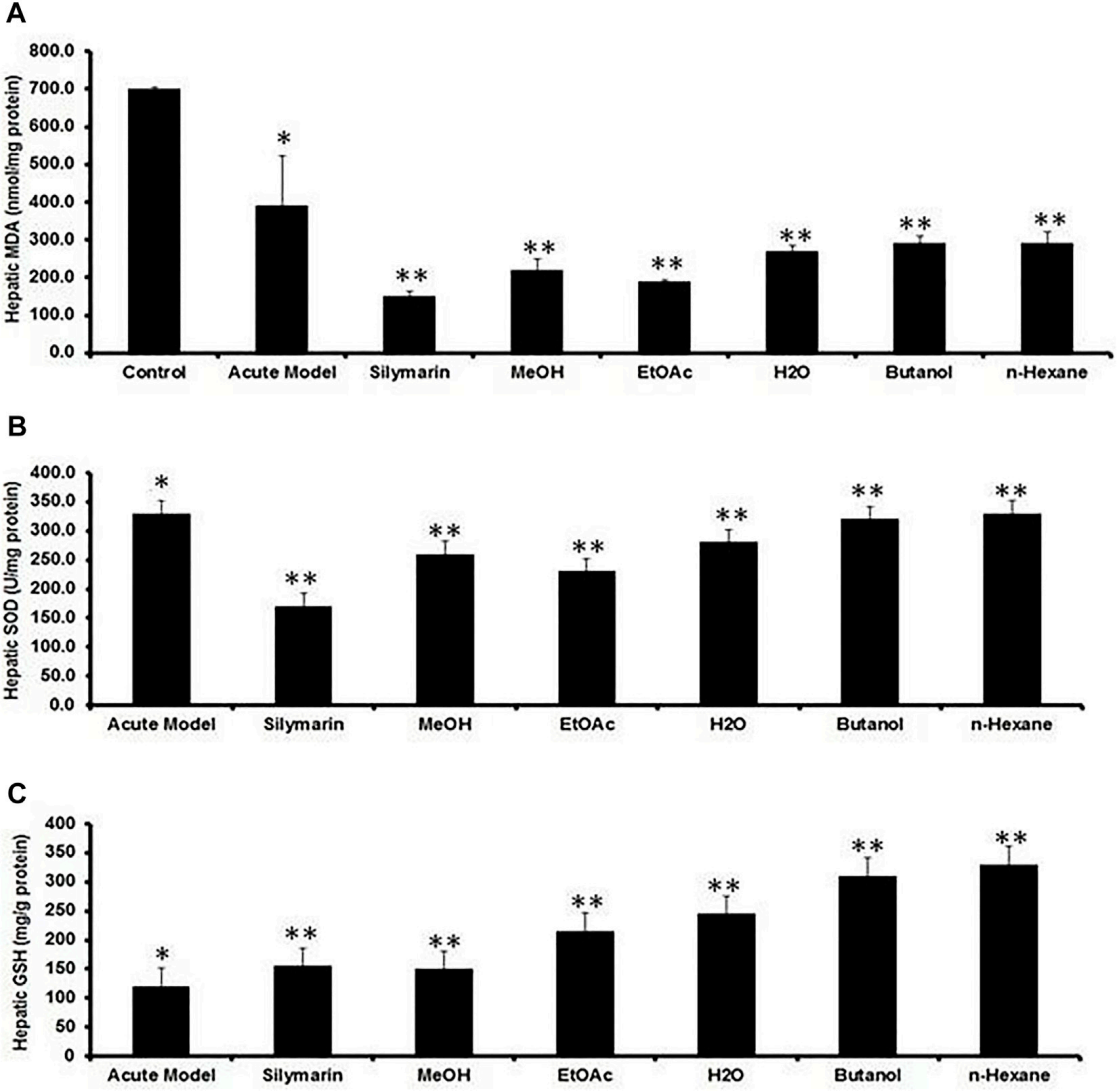
Quantification of the effects of *P. granatum* extract and fractions on antioxidant activity. **(A)** MDA, **(B)** SOD, and **(C)** GSH levels act as liver damage indicators under several conditions. The data were expressed as the mean ± SD where *p < 0.05, when compared to the normal control while MeOH extract and EtOAc and H_2_O fractions revealed best protection compared to CCl_4_ (**p < 0.001) and silymarin (**p < 0.001) treatment by increasing hepatic antioxidant enzymes and decreasing MDA levels, while the butanol and *n*-hexane fractions did not considerably elevate the hepatic antioxidant enzymes. Data were expressed as the mean ± SD where *n* = 10. PPx and its fractions mitigate the recruitment of Kupffer cells (KCs) during acute hepatotoxicity.

MDA is one of the main end products of lipid peroxidation, and its elevated level could be used as an indicator of hepatotoxicity. CCl_4_ intoxication significantly elevated the MDA content while reducing GSH and SOD levels in the liver tissues, compared to the normal control group (Figures 5A–C), indicating that the CCl_4_ injection induced severe oxidative stress. Treatment with Met-PPx and its EtOAc and aqueous fractions significantly attenuated this and were more effective than silymarin. Hepatic antioxidant enzyme levels were substantially increased (levels similar to those in the normal control) by Met-PPx and its EtOAc and aqueous fractions, which also significantly decreased MDA levels. In contrast, the butanol and the *n*-hexane fractions did not considerably increase the antioxidant activity and also showed high levels of MDA.

**FIGURE 6:**
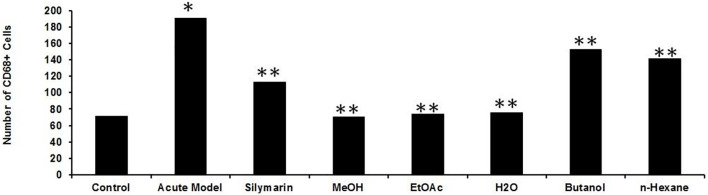
Effect of *P. granatum* fractions on the recruitment of Kupffer cells (KCs). Immunohistochemistry was performed for different groups: CCl_4_^+^ EtOAc fraction **(A–C)** or aqueous fraction **(D–F)**, butanol fraction **(G–I)**, and *n*-hexane fractions **(J–L)**. KCs that were positive **(A, D, G, J)** were presented by immunoreactivity. DAPI staining is shown in **(B, E, H, K)** for the nucleus, while the merged image is shown in **(C, F, I)**. The EtOAc **(A)** and H_2_O **(D)** fractions have abrogated the increase in Kupffer cells in hepatotoxicity, but the butanol **(G)** and *n*-hexane **(J)** fractions increase the number of KCs drastically. Scale bar is 50 μm.

**FIGURE 7:**
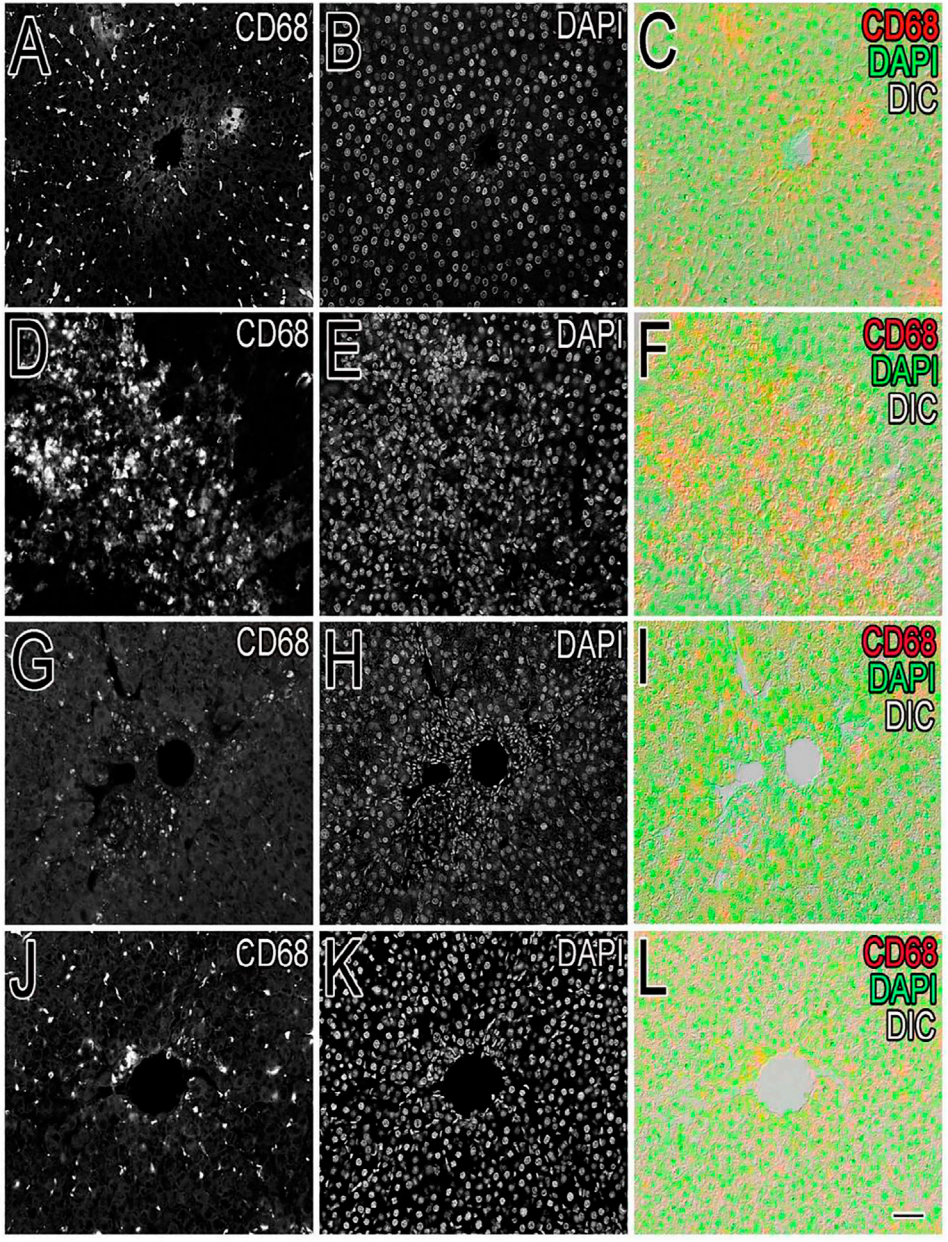
Quantification of the Kupffer cells after *P. granatum* extract treatment on CCl_4_-induced hepatotoxicity. After CCl_4_ intoxication resulting in membrane damage, activation of the dormant KCs occurred along with the higher number KCs compared to the normal control group (*p < 0.05). This increase was somewhat prevented by silymarin treatment (**p < 0.001), and while the Met-PPX (**p < 0.001), EtOAc (**p < 0.001), and aqueous fraction (**p < 0.001) significantly reduced KCs down to control levels, the butanol and *n*-hexane fractions did not considerably decreased their number. Immunostaining of inducible nitric oxide synthase (iNOS).

**FIGURE 8:**
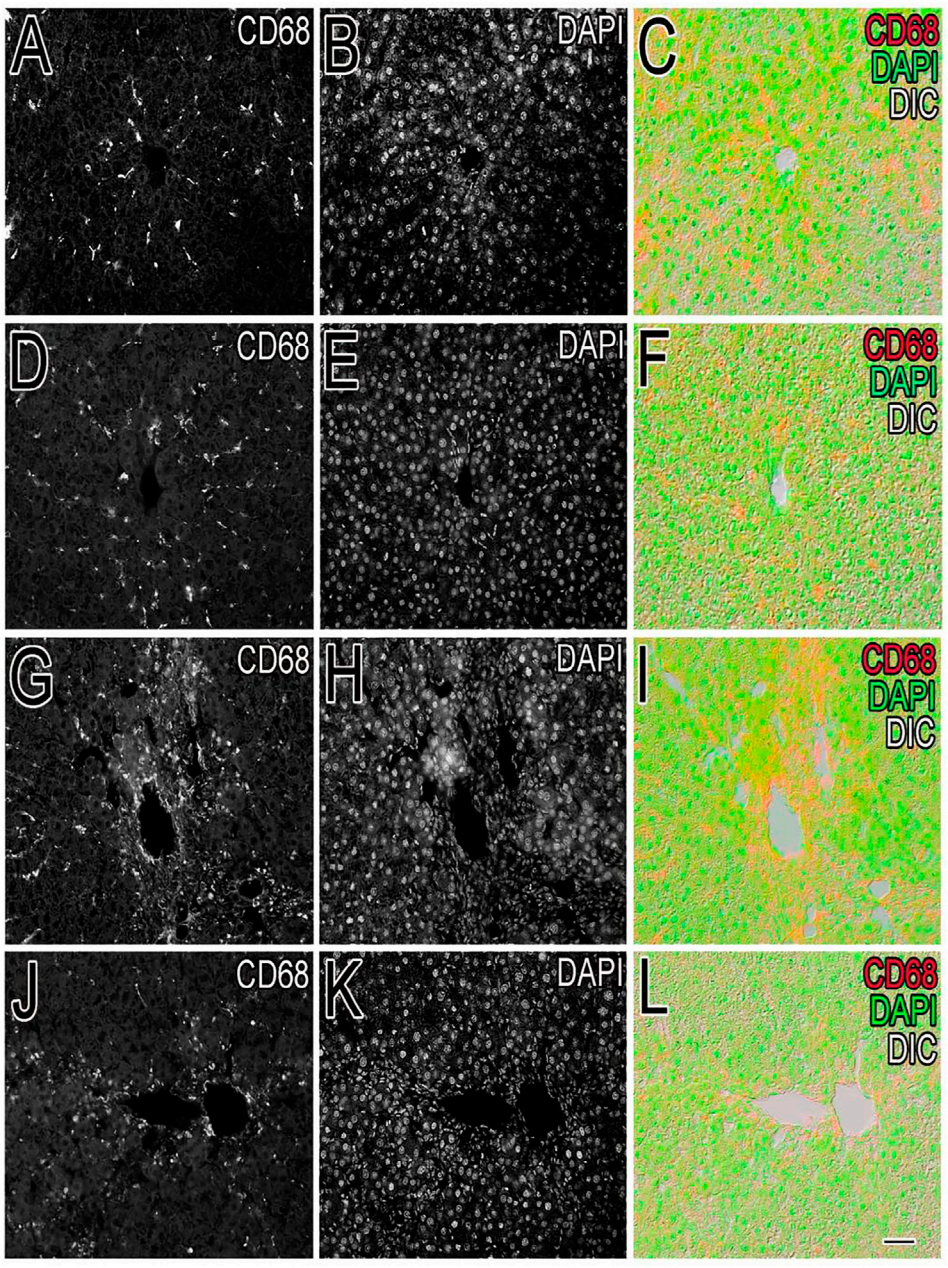
Effect of *P. granatum* extract on the recruitment of Kupffer cells (KCs). Immunostaining of KCs in different experimental groups was performed. The number of Kupffer cells **(A, D, G, J)** was examined in the normal control **(A–C)**, CCl_4_ **(D–F)**, silymarin-treated **(G–I)**, and Met-PPX **(J–L)** groups. DAPI was used for nucleus staining **(B, E, H, K),** while **(C, F, I, L)** shows the merge of DAPI + KCs. Considerably large number of KCs was found in the CCl_4_-intoxicated group **(D)** which is abridged by silymarin **(G)**. Interestingly, the Met- PPx **(J)** significantly reduced (*p* < 0.001) KCs around the central vein, similar to the normal control group. Scale bar is 50 μm.

Inflammation of the liver is linked with activation of Kupffer cells (KCs) and their migration into the hepatic cords where they secrete TNF- and IL-6, proinflammatory cytokines (Kiso et al., 2012). A particular KC marker, CD68, was used to monitor KC activation in CCl_4_-intoxicated rats. In the normal control liver (Figure 6), a small number (71 ± 10) of quiescent KCs are restricted to the liver sinusoids only (Figures 7A–C). The liver section of the control group did not show considerable CD68 immunopositivity (Figure 7A). In contrast, strong (*p* < 0.001) CD68 expression (190 ± 8) was observed around the central vein of the CCl_4_-intoxicated group (Figures 7D–F). CD68 immunoreactivity was observed mainly around the injured areas, presenting mostly pericentral staining. CD68 immunopositivity increased in the livers of CCl_4_-intoxicated rats. Mixed infiltration of inflammatory cells (Figure 7E) was identified from elongated nuclei with DAPI staining around the central vein in the CCl_4_-treated group. It is obviously known from Figure 6 that treatment with silymarin has decreased (*p* < 0.001) the number of Kupffer cells (112 ± 10) compared to the intoxicated group. Moreover, an enhancement in the number of KCs surrounding the central vein and the pericentral region in the liver treated with silymarin was still higher (*p* < 0.001), relative to the control group (Figures 7G–I). Interestingly, the Met- PPx significantly reduced (*p* < 0.001) KCs (71 ± 9) around the central vein (Figure 6), similar to the normal control group. After CCl_4_ intoxication, the activated KCs were recruited to the site of injury. The Met-PPx administration restricted the KCs to the sinusoidal spaces despite the CCl_4_ treatment as supported by the immunohistochemistry (Figures 7J–L). The presence of polyphenolic compounds in the EtOAc fraction might significantly reduce (*p* < 0.001) CD68-positive (74 ± 8) and limited the KCs to the sinusoids, similar to the Met-PPx group as evident from immunohistochemistry (Figures 7A–C). Likewise, the aqueous fraction completely restricted (*p* < 0.001) the KCs (75 ± 9) (Figure 6) to the sinusoids (Figures 8D–F). Compared with normal control sections, the butanol group revealed a drastic increase in the number of KCs (153 ± 11) (Figure 6) around the central vein (Figures 8G–I). Similarly, the *n*-hexane–treated group showed a dramatic increase (141 ± 12) in the number of KCs (Figures 6, 7J–LJ–L7J–L).

### Immunostaining of Inducible Nitric Oxide Synthase

**FIGURE 9:**
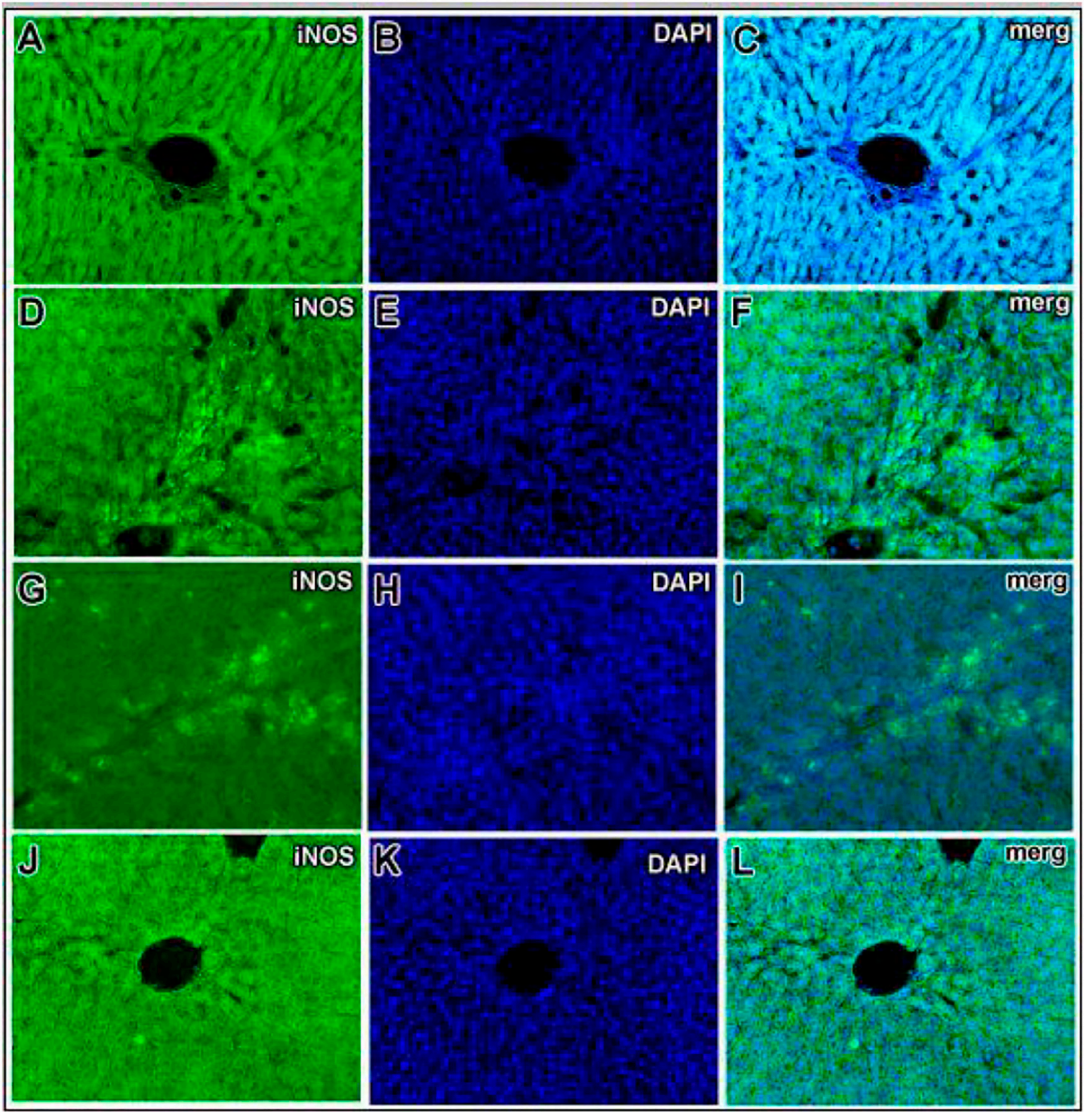
Hepatic inducible nitric oxide synthase (iNOS) expression in CCl_4_ **-**induced liver injury. iNOS immunoreactivity was almost absent in the normal control **(A–C)**. Prominent iNOS immunoreactivity in the livers of the CCl_4_-treated group **(D–F)** was observed. The CCl_4_-treated group showed strong iNOS immunopositive hepatocyte nuclei around the central vein region. Presence of immunopositive cells for iNOS in the silymarin-treated group **(G–I)**. In the 100 mg kg^−1^ ethyl acetate and aqueous–treated group, only minute traces of iNOS immunopositivity around the central vein hepatocytes and endothelial cells **(J–L)** were identified.

**FIGURE 10:**
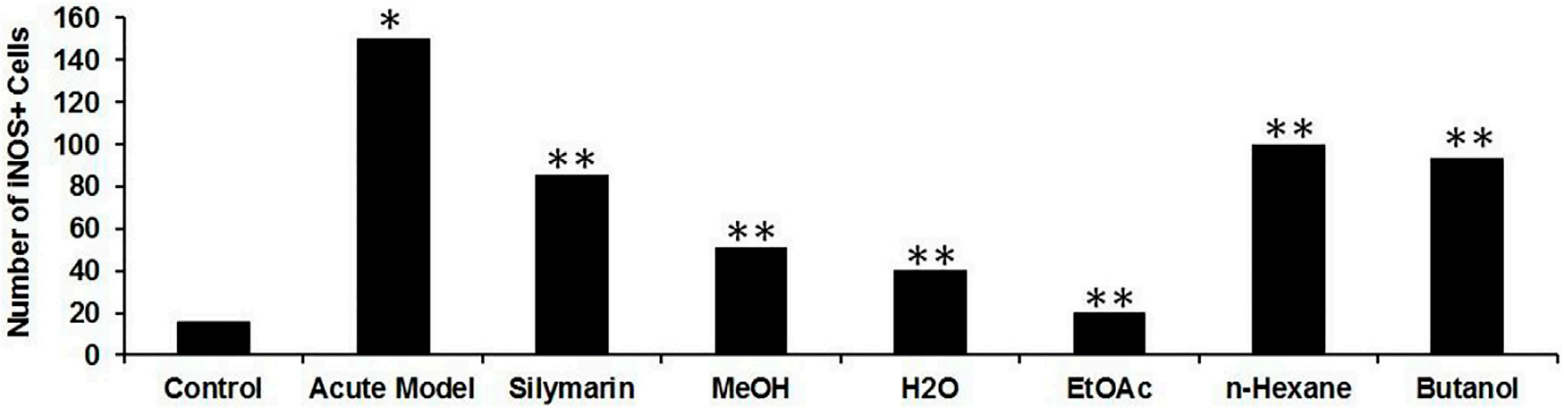
Quantification of iNOS expressed immunoreactive cells upon CCl_4_-induced hepatotoxicity. Injurious effect of CCl_4_ intoxication causing central lobular damage and with continuous expression of iNOS in the stressed hepatocytes (*p < 0.05) in the CCl_4_ group compared to the normal control. Due to the potential effect of silymarin, it was reduced somehow (**p < 0.001), and in contrast, the Met-PPX (**p < 0.001), EtOAc (**p < 0.001), and aqueous fraction (**p < 0.001) significantly abridged iNOS-positive immunostained cells to control levels, but in the *n*-hexane and butanol fractions, their number did not appreciably decline.

To investigate the role of hepatic iNOS *in vivo*, a CCl_4_-induced acute liver model of inflammation was used. In the control group, no apparent detection of iNOS expression was noticed by immunohistochemistry (Figures 9A,B), which was further confirmed from the merging of iNOS and DAPI as shown (Figure 9C). Moreover, quantification of the iNOS immunostained structure was identified and was measured under different conditions, showing a diminished number in contrast to the CCl_4_ group (Figure 10). After 24 h of administration of CCl_4_, the liver-associated necroinflammation is at its peak, especially ALT. In the CCl_4_-intoxicated model group, prominent expression of iNOS was observed and localized to the cytoplasmic content surrounding the hepatocytes’ nuclei (Figures 9D, 10). By double fluorescent microscopy, the enhanced expression and colocalization of iNOS and DAPI were uniformly confined in the surrounding central vein, which represents necrosis of hepatocytes (Figures 9E,F, 10). The positive control silymarin group also presented the expression of iNOS in the central and pericentral hepatocytes (Figures 9G, 10). The iNOS positively stained cells were scattered all around the central vein with signatures of necroinflammation (Figures 9H,I). In contrast, treatment with the ethyl acetate fraction showed negligible expression of iNOS (Figures 9J, 10), whereas iNOS expression was further reduced in the at 100 mg kg^−1^ in merged image (Figures 9L, 10). Moreover, hepatocyte and Kupffer cells’ nuclei were immune-negative for iNOS in the normal group like the ethyl acetate– and aqueous-treated groups.

## Discussion

The current study was undertaken to determine the possible hepatoprotective, antioxidant, and anti-inflammatory potential of the pomegranate peel extract (PPx) and its fractions in the CCl_4_-intoxicated rat model. Previous work has acknowledged the extensive pharmacological application of the pomegranate against a variety of diseases, particularly the effectiveness of its peel in abrogating the oxidative stress and curbing liver injury due to the presence of bioactive constituents. The antioxidant activity of the PG has been attributed to the presence of phenolic compounds having hydroxyl functional groups which act as potent hydrogen donors (Kulkarni et al., 2004). It has been suggested that peel powder and whey powder alone or synergistically exhibited antioxidant and hepatoprotective potential against CCl_4_-induced liver injury in rats (Ashoush et al., 2013). Another report suggested that the polysaccharides from pomegranate peel had significant antioxidant and hepatoprotective ability against CCl_4_-induced oxidative damage in mice (Zhai et al., 2018). In addition, the protective effects of pomegranate peel and seed extracts were demonstrated against CCl_4_-induced hepatic fibrosis (Wei et al., 2015). Pomegranate extracts and genistein have significant anticancer effect through growth inhibition in human breast cancer (Jeune et al., 2005). Although a few studies have focused on the isolation of pure compounds from PP and their antioxidant activities *in vitro* and *in vivo*, there is a lack of investigation on the involvement of KCs in hepatotoxicity. Downregulation of KCs may be one of the key factors to halt the progression of CCl_4_-induced hepatoxicity.

The CCl_4_-induced hepatotoxicity model is extensively used for investigating the mechanisms of hepatotoxic effects such as necrosis, apoptosis, fibrosis, and hepatocellular carcinoma and for hepatoprotective drug screening (Pierce et al., 1987). Normally the hepatic lobules consist of the central vein surrounded by hepatic cords of cells and sinusoids. CCl_4_ induces centrilobular necrosis of hepatocytes that can be detected by elevated levels of hepatic enzymes in the serum or histopathological study (Chen et al., 2017). It has been demonstrated previously that CCl_4_ is metabolized in the hepatocytes by cytochrome P450 enzymes into the CCl_3_ radical, which is further converted to the trichloromethyl peroxy radical in the presence of free oxygen. The highly reactive trichloromethyl peroxy radical subsequently binds to the polyunsaturated fatty acids of the cell membrane, resulting in peroxidative damage or stress. Oxidative stress plays a key role in the induction of hepatotoxicity by producing noxious lipid intermediates such as MDA and LPO that may interrupt the antioxidant defense, leading to hepatocyte necrosis (Brattin et al., 1985) and, ultimately, resulting in leakage and elevation of serum levels of enzymes, which is alleviated by the activities of SOD, GSH, and CAT (Kadiiska et al., 2005).

Elevation in the MDA content and levels of serum enzymes (ALT, AST, and ALP) and alleviated antioxidant enzyme activities, as evident in our study, indicates overproduction of free radicals due to failure of antioxidant defense mechanisms as a result of enhanced lipid peroxidation leading to loss of cell membrane integrity (Drotman and Lawhorn, 1978). Treatment with Met-PPx decreased the MDA and LPO levels by enhancing antioxidant activities of SOD, GSH, and CAT to attenuate CCl_4_-induced hepatotoxicity by reducing the oxidative stress. This extract also brought down the levels of AST, ALT, and ALP to corresponding normal values as compared to silymarin, which is a clue to the repair of hepatocyte damage and maintenance of the cell membrane and liver architecture. Likewise, EtOAC and the aqueous fractions enhanced the antioxidant activity and also alleviated the increase in serum enzymes. Nonetheless, the butanol and *n*-hexane fractions did not show satisfactory results. These biochemical findings were further supported by histopathological examination. These results suggest that the hepatoprotective effect of PPx and its fractions can be attributed to their antioxidative activities, also shown in previous studies (Singh et al., 2002).

Besides the aforementioned pathological aspects, inflammation is another important mechanism responsible for CCl_4_-induced hepatotoxicity (Huang et al., 2017). Inflammation is linked with the activation of KCs. Normally KCs reside in a quiescent state in the sinusoids. Oxidative stress triggers the release of TNF-α from damaged hepatocytes. The damaged hepatocytes activate and recruit the KCs into the injured area around the central vein, where they release proinflammatory cytokines, such as TNF-*α*, TGF-*β*, and IL-6. These cytokines in turn activate NF-κB and COX-2, responsible for the production of NO and prostaglandins, respectively, causing nitrosative stress and contributing to the augmentation of CCl_4_-induced hepatotoxicity (Kiso et al., 2012). We figured out that Met-PPx and its EtOAC and aqueous fractions overwhelm the inflammatory response by downregulating TNF-α and iNOS expression by attenuating oxidative stress and activation of KCs. Thus, the number of KCs around the central vein is reduced. However, butanol and *n*-hexane treatment showed a drastic increase in the number of KCs around the central vein, as evident from immunostaining, indicating that the active compound(s) is absent from these fractions.

## Conclusion

From the aforementioned results of the current study, it is concluded that Met-PPx and its EtOAC and aqueous fractions have hepatoprotective potential to normalize the changes in serum markers by raising the activity of the endogenous antioxidant defense mechanisms to preclude oxidative stress by quenching free radicals in CCl_4_-induced hepatotoxicity. Our findings also reveal that the Met-PPx and its EtOAC and aqueous fraction treatment significantly abrogated inflammatory response. Our work demonstrates that the hepatoprotective and free radical–quenching potential of the PP resides in the Met-PPx and its EtOAc and aqueous fractions due to the presence of bioactive compounds. Their use in precluding hepatotoxicity and ensuring the preserved structural integrity of the hepatocellular membrane deserves attention and further exploration.

## Data Availability

The raw data supporting the conclusion of this article will be made available by the authors, without undue reservation.
